# Evaluation of the accuracy of peri-operative CEUS in measuring the effect of microwave ablation treatment of uterine fibroids, in comparison with MRI

**DOI:** 10.1371/journal.pone.0356141

**Published:** 2026-08-25

**Authors:** Marie Beermann, Gudny Jonsdottir, Annika Cronsioe, Klara Hasselrot, Helena Kopp-Kallner

**Affiliations:** 1 Department of Radiology, Danderyd Hospital, Stockholm, Sweden; 2 Department of Clinical Sciences, Danderyd Hospital, Karolinska Institutet, Stockholm, Sweden; 3 Department of Obstetrics and Gynecology, Danderyd Hospital, Stockholm, Sweden; Chongqing Medical University, CHINA

## Abstract

**Objective:**

The objective of this study was to determine if peri-operative contrast enhanced ultrasound (CEUS) is as accurate as magnetic resonance imaging (MRI) in measuring the immediate effect of microwave ablation (MWA) treatment of uterine fibroids. Secondary outcomes were ablation ratio and proportion of recurrence of treated fibroids at 6 months.

**Methods:**

The study was a prospective observational trial. Seventeen patients with symptomatic uterine fibroids were included and treated with percutaneous ultrasound guided microwave ablation. Before the ablation, the volume of the up to three largest fibroids were recorded with MRI and CEUS. At the end of operation, the ablated volume was recorded with CEUS and compared to MRI within a week. At 6 months, the ablated volume was again recorded with MRI and any sign of regrowth of the treated fibroid(s) was noted.

**Results:**

A total of 19 fibroids were analyzed. The intraclass correlation coefficient (ICC) between ablation volume obtained by CEUS and MRI respectively was calculated to be 0.965 (95% confidence interval 0.909, 0.987). The Bland-Altman graph showed that the mean difference between volumes was –1.68 ml and standard deviation 22.60 ml, giving the limits of agreement (−45.98, 42.61). Regrowth of vascularized tissue was seen in 8 fibroids (47%).

**Conclusion:**

CEUS can be used to evaluate ablation volume and ablation ratio and is useful for intraoperative estimation and guidance. It shows strong correlation with MRI. Regrowth of fibroids may occur even when initial treatment was technically successful.

## Introduction

Uterine fibroids are common in women of reproductive age, reaching a lifetime incidence of up to 70% [1]. Although benign in nature, symptoms of heavy menstrual bleeding, pelvic pressure, pain and infertility may severely decrease quality of life in affected women [2]. Treatment recommendations should be individualized and based on symptoms, the size, number and location of fibroids, and desire for future pregnancy. If medical treatment is not sufficient, hysterectomy is often recommended as curative treatment but may lead to quite severe short- and long-term complications [3]. In recent years, increasing attention has been paid to minimally invasive therapies such as high-intensity focused ultrasound (HIFU), uterine artery embolization (UAE), radiofrequency ablation (RFA) and percutaneous microwave ablation (MWA), with focus on uterine preservation and symptom improvement [4]. HIFU is a totally non-invasive but time-consuming method, and best suited for women with fewer fibroids and smaller uteri [5]. Although non-invasive, bowel or other organs in the way of the ultrasound beam constitutes a contraindication to treatment [6]. UAE is an established method to decrease volume of fibroids and improve clinical symptoms [7], even though post-operative pain and post-embolization syndrome (PES) associated with the procedure have somewhat limited its use [8]. There has also been concern that UAE may increase the risk of earlier menopause by affecting ovarian function, and the risk appears to be greater in women older than 40 years. However, previous studies have yielded conflicting results [9,10]. RFA is a thermal ablation technique inducing coagulative necrosis of tissue around the tip of an antenna placed in the target of interest [11]. For treatment of fibroids, RFA has mainly been used with a laparoscopic or, of late, vaginal approach even though there are studies of percutaneous RFA showing promising results [12–14]. Like RFA, microwave ablation (MWA) generates heat and causes coagulative necrosis. Used correctly, it is safe, fast and easy to perform, and it is well tolerated by patients [15,16]. Compared to the other non- or minimally invasive thermal techniques discussed above, MWA has the advantage of consistently higher tissue temperatures leading to larger ablation volumes in shorter time. MWA is also consistently effective independent of tissue types, leading to a more predictable outcome [17].

To assess uterine fibroids in more detail, magnetic resonance imaging (MRI) has remained the gold standard [18]. However, in daily practice, and as treatment with thermal ablative techniques is increasingly performed by gynecologists, ultrasound is emerging as a method with obvious advantages such as possibility of pre-operative, peri-operative and post-operative evaluation [19]. In addition, MRI is both expensive and often scarce even in high resource countries [20] and it might pose logistical problems in an outpatient setting.

The microwave antenna is easily visible with ultrasound which allows for ultrasound guided placement using established methods for needle guidance. During the emission of microwaves, gray-scale ultrasound will show hyperechoic microbubbles starting from the emission point and spreading to cover the whole ablation zone [21]. In addition to gray-scale ultrasound, contrast enhanced ultrasound (CEUS) will show any residual vascularization of treated fibroids in real time and thus give the opportunity of repeated ablation if needed [21,22]. This is an important factor in improving ablation results.

Accuracy and generalizability of peri-operative CEUS have been established [22] but the aim of this study was to reproduce the results and to investigate if it can be used for accurate estimation of the ablation volume in a clinical setting where MRI is not available. Long-term follow up at 6 months was used to investigate the frequency of recurrence of ablated fibroids as a previous study from the same institution showed regrowth of successfully treated lesions [23], even though this is not very well described in the literature.

## Methods

The study was designed as a prospective observational trial. Seventeen consecutive women (willing to comply with protocol of the sub-study) who participated in a larger study at Danderyd Hospital, Sweden, from 01/01/2020 to 31/08/2023 were included. The parent study was aiming to evaluate the effect of ultrasound guided MWA of symptomatic uterine fibroids in a larger population.

All patients suffered from heavy menstrual bleeding, but many also had other symptoms such as pelvic pressure. They were premenopausal and had no wish for future pregnancies and were examined with vaginal ultrasound before inclusion. There was no limitation as to size, number and location of the fibroids. Included participants in the sub-study underwent contrast enhanced MRI before, within a week of ablation and again after 6 months, in addition to the ultrasound evaluation offered per protocol within the main study.

The ablation volume and the ablation ratio (calculated as the non-enhanced volume post-ablation divided by pre-ablation volume) measured by MRI were compared to the peri-operative CEUS (pre- and post-ablation). The ablation ratio was also correlated with the FIGO classification decided by MRI, as this has been shown to affect the treatment effect [5,14]. Long-term results were evaluated with MRI after 6 months, to investigate the frequency of recurrence of ablated fibroids. There was no CEUS performed at 6 months clinical follow-up, as it was not feasible in the gynecology office where the patients were seen.

The number of patients included in the sub-study was chosen to match a previous study from the same institution [24], as it was considered adequate for a feasibility study aimed at exploring the potential of CEUS for pre- and post-ablation assessment. An added inclusion criterium in the sub-study was willingness to comply with protocol including three MRI scans, and exclusion criterium was any condition entailing contraindication to contrast enhanced MRI. Suitable patients were informed orally and in writing by their responsible gynecologist and signed informed consent for the main study and the sub-study.

### MRI

For the MRI scans, a series of standard T1-weighted, T2-weighted and contrast enhanced T1-weighted imaging were performed with a 3T SIGNA^TM^ from GE HealthCare. On the pre-operative MRI, the individual volume of the up to three largest fibroids was recorded, as well as the vascularization (i.e., contrast enhancement, yes/no). Fibroid volume was calculated according to the formula width x height x length x 0.52. FIGO classification of the fibroids was used to categorize fibroid location into 3 groups: 1) FIGO 1–2: submucosal, 2) FIGO 3–5 including hybrid: intramural 3) FIGO 6: subserosal. Within a week and six months from the day of ablation, the ablated volume was recorded with MRI. At six months, any sign of regrowth of the treated fibroid(s) was noted. The MRI images were read by a senior consultant in radiology with 10 years of experience of gynecological MRI and if needed discussed with a second radiologist until a consensus agreement was reached.

### Ultrasound-guided MWA

Ultrasound (LOGIQ 10, GE HealthCare) was used for peri-operative imaging and needle guidance. Peri-operatively, the fibroid volume (width x height x length x 0.52) and vascularization were recorded with abdominal CEUS (2,4 ml SonoVue^®^, Bracco, Milan, Italy). At the end of the operation, the ablated (i.e., the non-perfused) volume was recorded in the same way and used for comparison with MRI within a week of ablation

With the patient under general anesthesia in the supine position the microwave antenna (Emprint™ Ablation Generator with Thermosphere™ Technology, Medtronic, Minneapolis, USA) was inserted into the fibroid percutaneously until the tip was 0.5–1 cm from the periphery of the fibroid. The distance was chosen depending on surrounding tissue, to avoid damage to heat-sensitive structures outside of the uterus. The microwave effect used was 60–100 Watts, depending on the size and location of the fibroid. The effect was monitored by ultrasound, and the ablation was stopped when the microbubbles, known to roughly represent the ablation zone, covered the whole fibroid or the part of the fibroid that was suitable for ablation. In larger lesions the antenna was pulled back and/or reinserted as necessary to cover the whole lesion. If CEUS showed signs of remaining vascularization in the treated fibroid, the procedure was repeated if safe.

The microwave ablations were carried out by an interventional radiologist with 10+ years of interventional experience.

### Statistical analysis

The agreement between ablated volumes per fibroid, obtained from CEUS and contrast enhanced MRI, was analyzed by using intraclass correlation coefficient (ICC) for absolute agreement and Bland-Altman regression. A p-value <0.05 was considered significant. Statistical analyses were performed using SPSS version 29 (IBM corporation, Armonk, New York, US).

### Ethics

The study was approved by the Regional Ethical Review Board of Stockholm (2019-04273 and Amendments 2020-00200 0327, 2020-01157).

## Results

**Fig 1:**
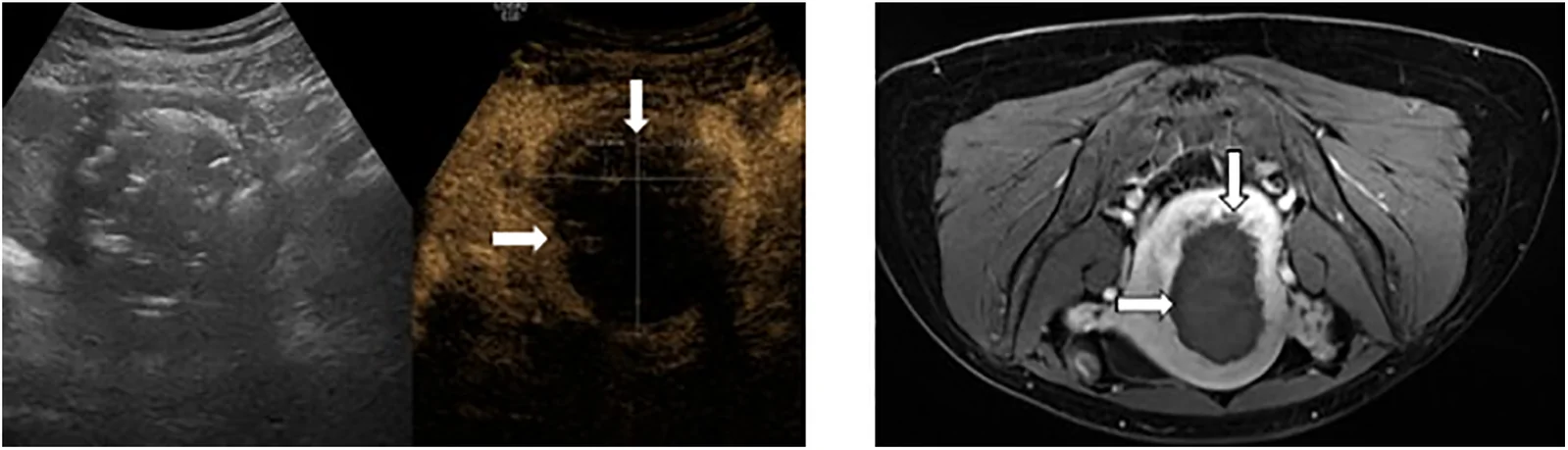
The avascular ablation zone marked by white arrows is seen as a dark area on contrast enhanced ultrasound (left) and contrast enhanced magnetic resonance imaging (right). For the ultrasound scan a transabdominal probe is used in the axial plane.

A total of 17 women with symptomatic fibroids were included in the sub-study. Two fibroids were treated in two women and as every lesion was considered as independent, a total of 19 fibroids were analyzed. The pre- and post-ablation volumes within a week measured on contrast enhanced MRI were compared with the results of the peri-operative CEUS. The mean time between ablation and post-operative MRI was 5.5 days, median 4 days. Fig 1 shows an example of what the ablation zones might look like with CEUS and MRI respectively. Two patients were lost to follow-up at 6 months. Age at first ablation (mean 45 years), number of ablated fibroids, FIGO classification and pre-ablation volumes are shown in Table 1. All fibroids were interpreted as being well vascularized pre-treatment, with MRI and CEUS alike. Four patients (24%) had fibroids that were not ablated due to small size or difficult location, in addition to the treated fibroid(s). The fibroids were treated with 60–100 Watts during 8–35.5 minutes, depending on size and location. Due to remaining vascularization on immediate post-operative CEUS, 6 fibroids out of 19 (32%) had an additional ablation in the same session. Table 2 shows the ablated volume and ablation ratio measured by CEUS and MRI. The mean of fibroid diameter and the mean volume before ablation was 6.56 + /- 1.63 cm and 156 + /- 102 ml respectively. The mean ablated volume (i.e., the avascular volume) was 104 + /- 60 ml (105 ml measured by MRI and 103 ml measured by CEUS). The mean ablation ratio (= volume of the non-enhanced field in the fibroid after ablation/ volume of the fibroid before ablation x 100) measured by ultrasound and MRI was 76% and 80%, respectively. The mean ablation ratio was 90%, 71% and 78% respectively for FIGO groups 1–3 which was not a significant difference, but the sample size is too small for generalization. For four fibroids the ablation volume was greater than the fibroid treated, due to ablation of some normal myometrium around the fibroid.

**Table 1:** Baseline characteristics and pre-operative volume of individual fibroids.

Pt.	Age at abl	Nr. fibroids treated	FIGO group	Pre-op volume MRI (ml)	Pre-op volume CEUS (ml)
1	47	1	1	129	155
2	46	1	1	23	22
		2	2	298	224
3	47	1	2	256	259
4	47	1	1	139	130
5	51	1	2	147	121
6	48	1	1	52	88
7	46	1	3	210	201
8	47	1	1	134	138
9	42	1	1	116	68
10	40	1	2	254	292
11	38	1	2	415	326
12	48	1	2	224	261
13	44	1	1	123	132
14	46	1	3	189	144
15	39	1	2	53	60
16	49	1	1	83	87
		2	2	7	6
17	43	1	1	110	97

* Pt=patient.

† Abl = ablation.

‡ FIGO group 1: submucosal, FIGO group 2: intramural (including hybrid), FIGO group 3: subserosal.

**Table 2:** Ablation volume and ratio measured by CEUS and MRI and signs of regrowth of ablated fibroid.

Pt.	FIGO group	Abl volume CEUS	Abl volume MRI	Abl ratio CEUS (%)	Abl ratio MRI (%)	Abl volume MRI 6m	Regrowth 6m
1	1	86	115	56	89	2	+
2	1	27	23	123	100	6	–
	2	172	109	77	37	49	+
3	2	107	109	41	43	65	–
4	1	115	109	88	78	Missing	Missing
5	2	71	73	59	50	43	–
6	1	75	105	85	202	8	–
7	3	149	154	74	73	104	–
8	1	97	95	70	71	15	+
9	1	55	57	81	49	7	+
10	2	253	254	87	100	132	–
11	2	203	205	62	49	85	+
12	2	117	155	45	69	Missing	Missing
13	1	118	101	89	82	82	–
14	3	130	156	90	83	25	–
15	2	30	32	50	60	10	+
16	1	49	59	56	71	10	+
	2	6	11	100	157	2	–
17	1	100	70	103	64	6	+

* Pt=patient.

† Abl=ablation *Abl=ablation.

‡ CEUS = contrast enhanced ultrasound.

§ MRI = magnetic resonance imaging.

¶ W = Watts.

**Fig 2:**
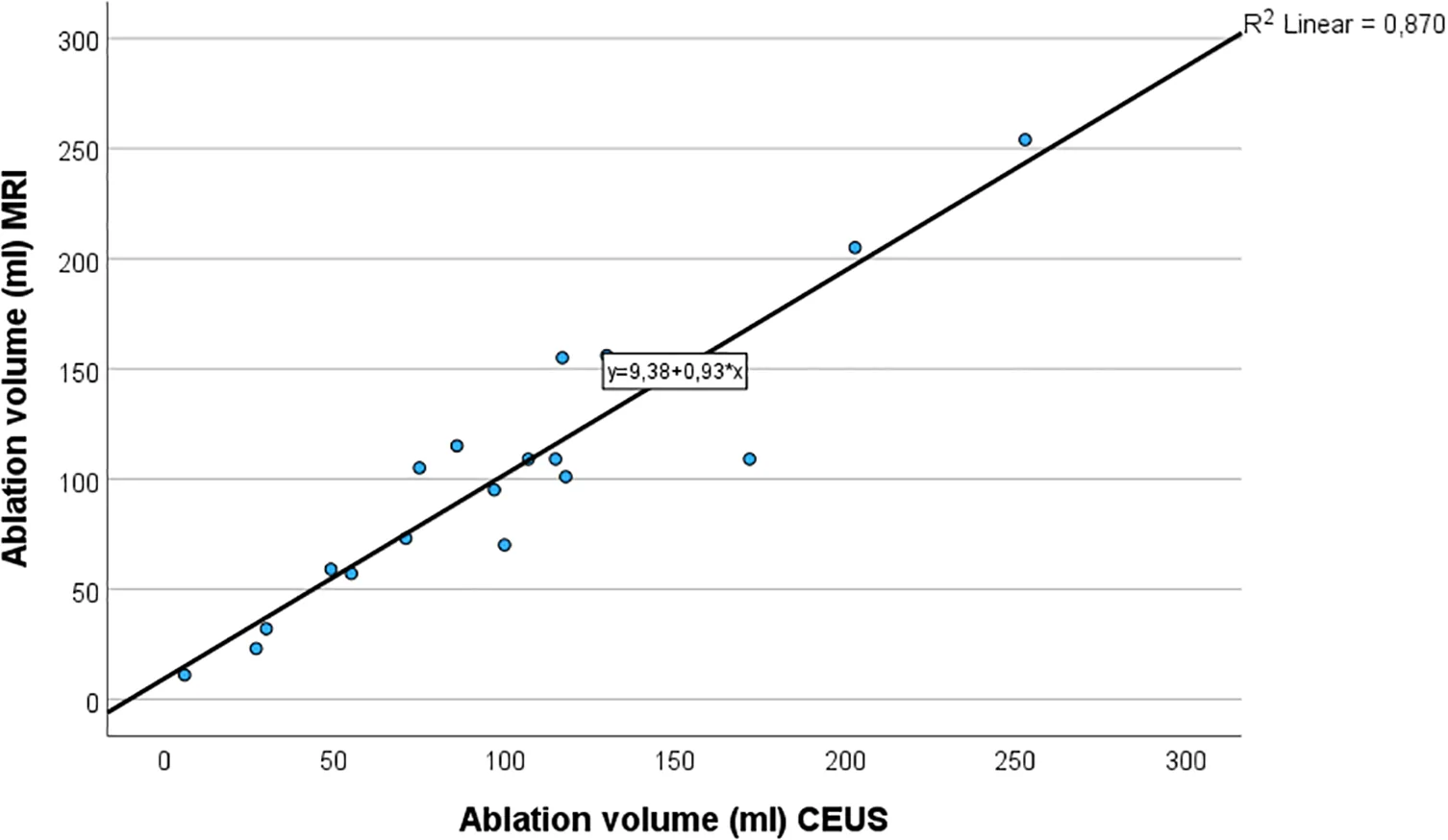
Ablation volumes (ml) obtained from contrast enhanced ultrasound and contrast enhanced magnetic resonance imaging, with regression line. The interclass correlation coefficient (ICC) was 0.965 and the agreement is considered good if it is > 0.75.

The volumes obtained from CEUS and MRI were plotted and a regression line was drawn indicating a linear trend between the two groups of measurements (Fig 2). The intraclass correlation coefficient (ICC) was calculated to be 0.965 (95% confidence interval 0.909, 0.987).

**Fig 3:**
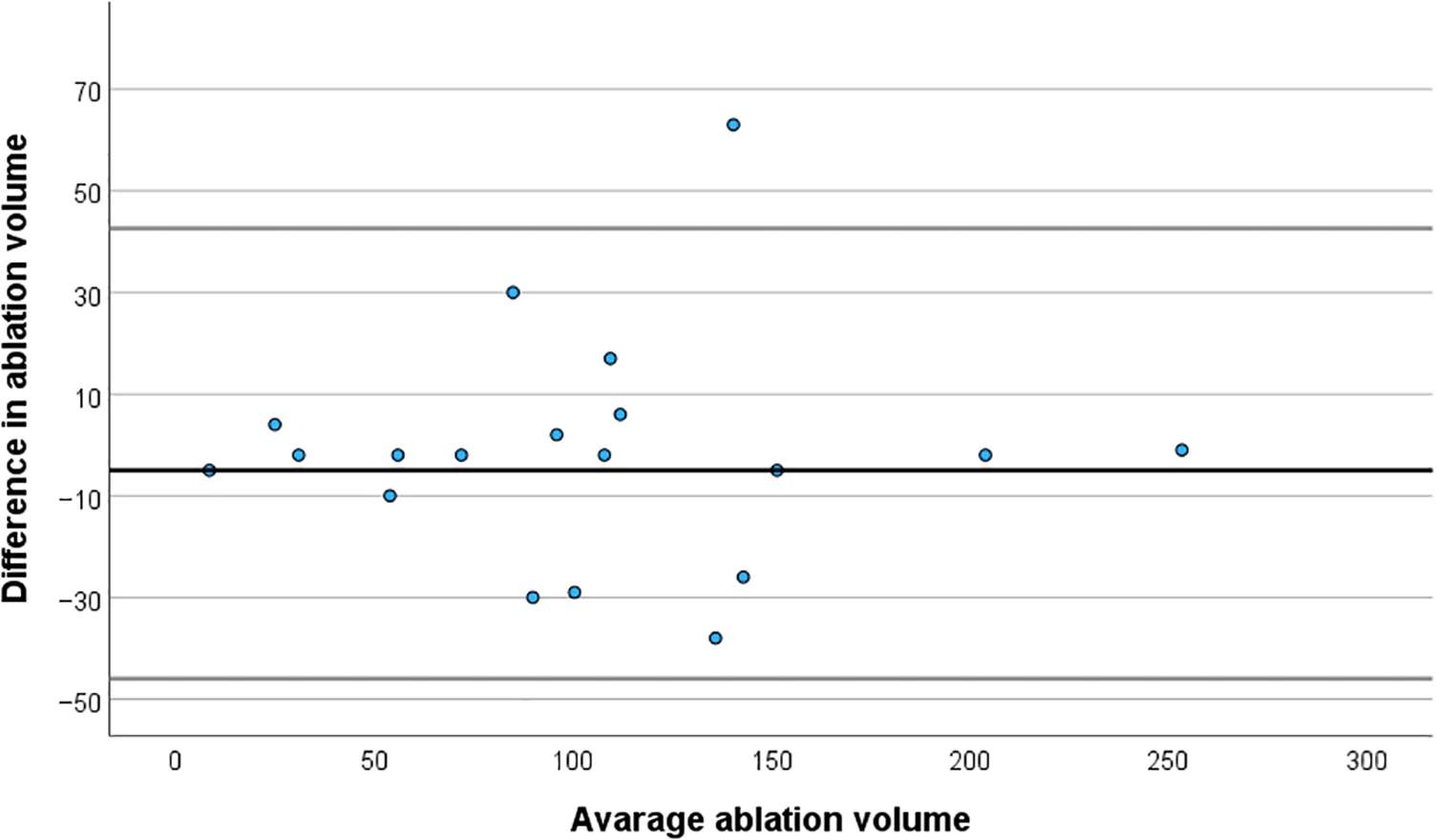
Bland-Altman plot, with 95% limits of agreement (grey lines). The black line constitutes the mean.

The difference between, and the mean of, ablation volumes measured by CEUS (immediately after ablation) and MRI (within a week from ablation) were calculated, and correlation (difference, mean) was 0.008 (p = 0.932), indicating that the difference is independent of the mean and that no proportional bias is present. The data could therefore be used for the Bland-Altman graph plotted in Fig 3. The mean difference between volumes was –1.68 ml and standard deviation 22.60 ml, giving the limits of agreement (−45.98, 42.61). The graph shows that all points but one fall within the 95% limit of agreement. The maximum difference between the groups (ablation volumes measured by CEUS and MRI) was 63 ml. Within the limits of agreement, the largest difference was 38 ml.

The MRI follow-up at 6 months showed that 17 out of 19 (89%) of the ablation zones had decreased in size as expected. Regrowth of vascularized fibroid tissue, defined as recurrent contrast enhancement in treated lesions, was seen in 8 fibroids (47.1%, 95% CI: 26.2%–69.0%).

## Discussion

The aim of this study was to investigate if the use of contrast enhanced ultrasound (CEUS) in the treatment of microwave ablation of uterine fibroids can be used as an alternative to MRI for evaluation of the ablation volume, at the time of the procedure. Even though MRI is the gold standard for imaging of fibroids, it is a bottleneck in many countries and hospitals, in addition to its high cost and obvious disadvantages such as an impossibility of using it for intra-operative evaluation. Thus, in the mother study of 123 women, ultrasound alone was used for pre- peri- and post-ablation monitoring to save time and money [25]. Using CEUS for immediate evaluation in a peri-operative setting enables visualization of any remaining vascularization within treated fibroids which can then be complimentary ablated in the same session. In our study, 6 fibroids out of 19 (32%) had an immediate additional ablation after the evaluation with CEUS. This must be considered a major advantage of CEUS over MRI. Our results, as presented in Table 2, are in agreement with previous studies from China. In a study from 2014, Lei et al. [22] found good correlation between ablation volumes measured by CEUS and post-operative contrast enhanced MRI, and Zhang et al. [26] concluded that CEUS could even be advantageous over MRI as it gives reliable real-time information on the perfusion and possible viable fibroid residue within the ablated area. Our study confirms these results in a different setting adding to the generalizability of the method, which is of importance before implementing it in a clinical practice outside of Asia.

The agreement between ablated volumes obtained from CEUS and contrast enhanced MRI was analyzed by the intraclass correlation coefficient (ICC) and Bland-Altman regression.

The intraclass correlation coefficient (ICC) was calculated to be 0.965 (95% confidence interval 0.909, 0.987). The agreement can be considered as good if ICC is higher than 0.75 [27]. The Bland-Altman analysis is used to evaluate the agreement between two measurement techniques. It allows for identification of any systematic difference or possible outliers and can help compare a new measurement method to a reference standard. In our study the mean difference between volumes was –1.68 ml and the standard deviation 22.60 ml, giving the limits of agreement (−45.98, 42.61) as depicted in Fig 3. Within the limits of agreement, the largest absolute difference was 38 ml. It is a larger difference (36.5% of the mean ablated volume) than in a similar study by Lei et al. (11.8%) [22]. This could have many explanations, one being size. The mean diameter of treated fibroids in this study was 6.56 + /- 1.63 cm and mean volume was 156 ml before ablation. This is higher than in the study by Lei et al (mean diameter 5.56 + /-1,26 cm, mean volume not specified). The largest difference between the ablation volume measured by CEUS and MRI in this study was 63 ml in a clear outlier. It was noted in the second largest fibroid with a pre-operative volume of 298 ml (MRI). For large lesions, ultrasound may have the limitation of smaller field of view than MRI. In addition, a characteristic of fibroids is echo-shadowing, which further complicates the measurement of large fibroids and for these lesions MRI is a more precise method. Due to technical reasons, it is also difficult to achieve spherical ablation zones larger than 5–6 cm in diameter [28], and for very large fibroids other treatments can be considered as more appropriate. Also, the bubbles present in the ablation zone immediately after treatment might obscure the ultrasound image and make correct measurements more difficult to obtain. This could be avoided by delaying the post-operative ultrasound, but in clinical practice with a patient under sedation or general anesthesia this is usually not an option and would also prevent the possibility of repeated treatment of residual areas of vascularization within the fibroid in the same session, as discussed above. For logistical reasons, the post-operative MRI could not be performed immediately following the ablation. The mean time between ablation and post-operative MRI was 5.5 days (median 4 days). A study of microwave ablation of liver tumors by Alzubaidi et al. [29] found that the ablation zone expanded during the first 24 hours after treatment, likely due to evolving edema and tissue response. None of our patients had the post-operative MRI performed within the first 48 hours. Thus, remaining post-operative expansion of the non-enhancing zone is not likely to affect results but as discussed previously, large lesions are difficult to evaluate with ultrasound. For most cases (68%), the absolute difference between volume measured by CEUS and MRI was less than 10 ml and in conclusion we argue that the CEUS evaluation can be performed intra-operatively and that the two methods agree sufficiently to support the use of CEUS in our clinical setting of outpatient treatment.

If CEUS replaces MRI for evaluation of MWA treatment of uterine fibroids, adequate training of the provider in use of CEUS is necessary We used a commercially available product for CEUS and a well-established technique for fibroid evaluation. We therefore believe that our results are generalizable to any setting with corresponding experience with ultrasound evaluation with CEUS.

In countries where CEUS is not available, gray-scale ultrasound will be able to assist the ablation treatment but not to reliably assess the avascular ablation zone.

The ablation ratio was high at 76–80% (measured by ultrasound and MRI, respectively). Notably, not all fibroids can be completely ablated safely due to location with heat sensitive tissues in close proximity to the fibroid, but a reduction of vascularized volume may still reduce symptoms, as shown in previous studies [25]. In ablation of malignant liver tumors, minimal ablative margin (MAM) is important for improving local control following thermal ablation [30]. At least 5 mm of healthy tissue surrounding the tumor should be covered by the ablation zone, to reduce the risk of local recurrence. When it comes to benign lesions like fibroids safety comes first, but if there are no heat sensitive structures at risk the aim should be complete ablation. For four fibroids the ablation ratio exceeded 100%. All were classified as FIGO group 1 (submucosal) which, due to location, allowed more aggressive treatment and thus an ablation zone covering more than the actual lesion. More commonly in this study, the outermost rim of the fibroid was left to avoid harm.

Four out of 17 patients (24%) had more fibroids than the 1–2 treated, and depending on the woman’s age at time of treatment these lesions can potentially grow to symptomatic size over time [31]. Also, in almost 50% of ablated patients (47.1%, 95% CI: 26.2%–69.0%) there was regrowth of vascularized fibroid tissue defined as recurrent contrast enhancement in treated lesions, even though the ablation volume (i.e., the avascular part of the fibroid) continued to shrink. The wide confidence interval due to the small sample size makes it difficult to generalize the results, but we proposed that when a rim of the fibroid must be left untreated due to the safety of surrounding structures as discussed above, there is a potential for regrowth. The same results were shown in a previous study from the same institution [23] but has not, to our knowledge, been well described in the literature. For tumors treated with thermal ablation in other organs, re-ablation of local recurrence is an established method [32,33]. We propose that re-ablation could be an option also for uterine fibroids showing signs of regrowth or inadequate ablation results, especially since the patient acceptance of the method is high [24]. Treatment methods like uterine artery embolization (UAE) may be as effective [24] but not as well tolerated as MWA [34]. Our intention is to investigate the efficacy of reablation further in future studies.

A limitation to the study is the lack of blinding and inter-/intra-observer reliability assessment of imaging (CEUS and MRI). However, as the volumes measured by CEUS and MRI were compared after all data were collected and there was an option of discussion among colleagues until consensus was reached, we believe the effect on the results is limited.

Another general limitation to the study is the small number of cases. It is to be considered as a feasibility study to evaluate if intraoperative CEUS is a useful method for pre- and post-evaluation of microwave ablation of fibroids in an outpatient setting, without the support of MRI and outside of Asia. Also, even with the current sample size, the correlation between CEUS and MRI was strong enough to support CEUS for intraoperative estimation and guidance.

In conclusion, we believe that ultrasound and specifically CEUS could replace MRI for evaluation of the ablation zone in a clinical setting. Microwave ablation seems to be an effective method for treatment of symptomatic fibroids with a mean ablation rate of 76–80% in this study. The 6-month follow-up indicates that regrowth of fibroids will occur even when initial treatment was technically successful, suggesting that re-ablation could be considered.
