# Supplementary material for: Evaluation of the accuracy of peri-operative CEUS in measuring the effect of microwave ablation treatment of uterine fibroids, in comparison with MRI
Source: PLoS One. 2026 Aug 25;21(8):e0356141. doi: 10.1371/journal.pone.0356141 (PMC13505896; doi:10.1371/journal.pone.0356141)
Supplement: Supplementary file 1 [file pone.0356141.s002.docx]

**Response to Reviewers**

Thank you so much for taking the time to review this manuscript. We appreciate the chance to improve our work according to your remarks! Below, our response is in **bold print.** The line references correspond to the Revised Manuscript with Track Changes.

Journal Requirements:

1. Please ensure that your manuscript meets PLOS ONE's style requirements, including those for file naming.

**Thank you, we have tried to change the manuscript files according to your requirements.
Note that Fig. 1 constitutes two images saved in the required format as Picture1a and Picture1b. The complete figure, as created by the author, is also included as a template.**

2. We note that the grant information you provided in the ‘Funding Information’ and ‘Financial Disclosure’ sections do not match.

When you resubmit, please ensure that you provide the correct grant numbers for the awards you received for your study in the ‘Funding Information’ section.

**Checked and changed.**

3. Please note that funding information should not appear in any section or other areas of your manuscript. We will only publish funding information present in the Funding Statement section of the online submission form. Please remove any funding-related text from the manuscript.

**Thank you, this information has been removed from the manuscript.**

4. Thank you for stating the following in the Competing Interests section:

“Marie Beermann reports a relationship with Medtronic that includes non-financial support and travel reimbursement.

Helena Kopp Kallner reports a relationship with Medtronic that includes non-financial support and travel reimbursement. Helena Kopp-Kallner reports a relationship with Mermaid Medical that includes non-financial support.

Other authors declare that they have no known competing financial interests or personal relationships that could have appeared to influence the work reported in this paper.”

Please confirm that this does not alter your adherence to all PLOS ONE policies on sharing data and materials, by including the following statement: "This does not alter our adherence to PLOS ONE policies on sharing data and materials.” (as detailed online in our guide for authors <http://journals.plos.org/plosone/s/competing-interests>). If there are restrictions on sharing of data and/or materials, please state these. Please note that we cannot proceed with consideration of your article until this information has been declared.

Please respond by return email with your amended Competing Interests Statement and we will change the online submission form on your behalf.

**Thank you! It has been done.**

5. Please provide a complete Data Availability Statement in the submission form, ensuring you include all necessary access information or a reason for why you are unable to make your data freely accessible. If your research concerns only data provided within your submission, please write "All data are in the manuscript and/or supporting information files" as your Data Availability Statement.

**Thank you, we are providing this data availability statement:**

**Data is provided as in the manuscript. More detailed data including the full dataset can be shared within collaborative efforts after signing agreements for data sharing and/or transfer. Data containing personal information cannot be shared without agreement, according to the General Data Protection Regulation (GDPR).**

6. If the reviewer comments include a recommendation to cite specific previously published works, please review and evaluate these publications to determine whether they are relevant and should be cited. There is no requirement to cite these works unless the editor has indicated otherwise.

**Not applicable.**

Reviewers' comments:

Reviewer #1: This study addresses a clinically meaningful question — whether intraoperative CEUS can replace MRI for evaluating microwave ablation of uterine fibroids — and the methodological approach is reasonable in its general design. However, the manuscript has several fundamental limitations that prevent the data from supporting the conclusions as currently stated.

1. Interchangeability is not established
The primary conclusion that CEUS and MRI "can be used interchangeably" is not justified by the data. The Bland-Altman limits of agreement span 88.6 ml (−45.98 to 42.61 ml), representing approximately 85% of the mean ablated volume. No minimum clinically important difference (MCID) was prespecified, making it impossible to determine whether this range of disagreement is clinically acceptable. The authors are reminded that a high ICC reflects rank-order consistency, not method interchangeability; the two must not be conflated.

**We thank you for this meaningful comment. This has been changed and/or addressed in the Abstract (line 38-39), the Introduction (line 78-79) and the Discussion (327-329) and the word interchangeable is no longer used in the manuscript.**

**The text now reads:**

**Abstract: CEUS can be used to evaluate ablation volume and ablation ratio and is useful for intraoperative estimation and guidance. It shows strong correlation with MRI.**

**Introduction: Accuracy and generalizability of peri-operative CEUS has been established [12] but the aim of this study was to reproduce the results and to investigate if it can be used for accurate estimation of the ablation volume in a clinical setting where MRI is not available.**

**Discussion: Also, even with the current sample size, the correlation between CEUS and MRI was strong enough to support CEUS for intraoperative estimation and guidance.**

2. Timing bias is inadequately addressed
CEUS was performed intraoperatively, while MRI was obtained within one week of ablation. Post-ablation inflammatory edema may systematically expand the non-enhancing zone visible on MRI, introducing a directional bias into the volume comparison. The authors briefly mention acoustic bubble interference as a limitation of CEUS but do not analyze or quantify the potential effect of this timing difference. The actual median interval between procedures should be reported, and its implications for the results must be discussed.

**We agree that information about timing of the post-operative MRI is missing and have added the median interval between ablation and MRI (Results; line 161-162) and the implication of this timing is further discussed (Discussion; line 274-285).**

**The text now reads:**

**Results: The mean time between ablation and post-operative MRI was 5,5 days, median 4 days.**

**Discussion: For logistical reasons, the post-operative MRI could not be performed immediately following the ablation. The mean time between ablation and post-operative MRI was 5,5 days (median 4 days). A study of microwave ablation of liver tumors by Alzubaidi et al. [19] found that the ablation zone expanded during the first 24 hours after treatment, likely due to evolving edema and tissue response. None of our patients had the post-operative MRI performed within the first 48 hours. Thus, remaining post-operative expansion of the non-enhancing zone is not likely to affect results but as discussed previously, large lesions are difficult to evaluate with ultrasound. For most cases (68%), the absolute difference between volume measured by CEUS and MRI was less than 10 ml and in conclusion we argue that the CEUS evaluation can be performed intra-operatively and that the two methods agree sufficiently to support the use of CEUS in our clinical setting of outpatient treatment.**

**However, we do not agree that the ablation zone will continually expand after 24 h, and as none of the patients did an MRI within 48 h we do not believe that the timing is the reason for the differences in volume (but rather the difficulty of measuring large lesions with ultrasound, as discussed).**

3. No sample size justification
No a priori sample size calculation is provided for either the primary or secondary outcomes. Matching the sample size of a prior study from the same institution does not constitute a methodological justification. The authors should either provide a formal power calculation or explicitly frame this as a feasibility study, with conclusions worded accordingly.

**We agree. As this was an exploratory study, no power calculation was performed for this outcome. We have clarified this in Methods; line 104-106.**

**The text now reads: The number of patients included in the sub-study was chosen to match a previous study from the same institution [14], as it was considered adequate for a feasibility study aimed at exploring the potential of CEUS for pre- and post-ablation assessment.**

4. Secondary outcomes are statistically underpowered
The recurrence rate of 47% is based on 8 events among 15 patients with complete follow-up. The corresponding 95% confidence interval spans approximately 21% to 73% — far too wide to be clinically informative — yet no confidence interval is reported in the manuscript. The subgroup analysis by FIGO classification includes only 2 fibroids in the subserosal group; reporting a non-significant difference across groups at this sample size risks being misinterpreted as evidence of equivalence.

**Thank you for pointing this out. However, we do feel that it is clinically important to point out recurrences, as these are seldomly transparently reported in the literature. We have added a line addressing this (Results; line 178) and deleted the lines 228-230 (... no signs of regrowth were seen in the 2 subserosal fibroids included in the study.)**

**Added line: ...but the sample size is too small for generalization.**

5. Ablation ratio data are internally inconsistent
The text states that ablation volume exceeded fibroid volume in two cases. Table 2 shows ablation ratios exceeding 100% in more than two cases (e.g., Patient 2 CEUS 123%, Patient 16 MRI 157%). This discrepancy must be fully reconciled, with case-by-case explanation provided for all instances where the ratio exceeds 100%.

**Thank you for bringing this up as it needed clarification. It is always difficult to tell exactly where the original tumor is localized within an ablation zone, hence we chose to measure the whole avascular area. This is further discussed in Discussion; line 299-307.**

**The text now reads: In ablation of malignant liver tumors, minimal ablative margin (MAM) is important for improving local control following thermal ablation[20]. At least 5 mm of healthy tissue surrounding the tumor should be covered by the ablation zone, to reduce the risk of local recurrence. When it comes to benign lesions like fibroids safety comes first, but if there are no heat sensitive structures at risk the aim should be complete ablation. For four fibroids the ablation ratio exceeded 100%. All were classified as FIGO group 1 (submucosal) which, due to location, allowed more aggressive treatment and thus an ablation zone covering more than the actual lesion. More commonly in this study, the outermost rim of the fibroid was left to avoid harm.**

6. Additional concerns
The recurrence outcome ("regrowth of vascularized tissue") lacks an operational definition. Whether this refers to new contrast enhancement within the ablation zone or an increase in overall fibroid volume should be specified, as the distinction carries different clinical and pathophysiological implications.

**Thank you! We have added this under Results; line 227-228 and Discussion; line 311-315.**

**The text now reads:**

**Results: Regrowth of vascularized fibroid tissue, defined as recurrent contrast enhancement in treated lesions, was seen in 8 fibroids (47%).**

**Discussion: Also, in almost 50% of ablated patients there was regrowth of vascularized fibroid tissue defined as recurrent contrast enhancement in treated lesions, even though the ablation volume (i.e. the avascular part of the fibroid) continued to shrink. When a rim of the fibroid must be left untreated due to the safety of surrounding structures as discussed above, there is a potential for regrowth.**

The CEUS volume calculation method is never described. MRI uses the ellipsoid formula (length × width × height × 0.52); it is unclear whether the same formula was applied to CEUS measurements.

**We apologize. It has been added under Methods; line 127 (width x height x length x 0.52).**

Neither blinding nor inter- or intra-observer reliability is reported for either modality. Given that MRI serves as the reference standard, its measurement reproducibility should be established.

**Thank you for addressing this, which is clearly a weakness of our study. We have touched on this under Methods; line 123 (“discussed with a second radiologist until a consensus agreement was reached”). We did not have the resources to do a proper inter-observer reliability study.**

The ICC model specification (one-way vs. two-way; absolute agreement vs. consistency; single vs. average measures) is not reported and must be added.
One data point in Figure 3 visually exceeds the upper limit of agreement but is not identified or discussed in the text. This outlier should be labeled and addressed.

**A clarification of the ICC model has been added to Statistical analysis; line 148-149. It should be one-way, absolute agreement.**

**It is true that there was one clear outlier. This text was included in the results section: The graph shows that all points but one fall within the 95% limit of agreement (line 218-219).** **We have added information on this in the discussion.**

**The text now reads: The largest difference between the ablation volume measured by CEUS and MRI in this study was 63 ml in a clear outlier. It was noted in the second largest fibroid with a pre-operative volume of 298 ml (MRI). For large lesions, ultrasound may have the limitation of smaller field of view than MRI. In addition, a characteristic of fibroids is echo-shadowing, which further complicates the measurement of large fibroids and for these lesions MRI is a more precise method Due to technical reasons it is also difficult to achieve spherical ablation zones larger than 5-6 cm in diameter [18], and for very large fibroids other treatments can be considered as more appropriate.**

Reviewer #2: Review of PONE-D-26-16095

Evaluation of the accuracy of peri-operative CEUS in measuring the effect of microwave ablation treatment of uterine fibroids, in comparison with MRI

Summary and general comments
The authors present results from a small study in 19 women using CEUS and MRI to measure ablation volumes and outcomes after MWA of uterine fibroids. The authors found an R2 value of 0.97 between CEUS and MRI volumes. CEUS enabled immediate retreatment in 6 cases. Overall 47% of the fibroids showed recurrence after one year. The is an interesting manuscript, but the excellent agreement between CEUS and MRI is somewhat understated and the Discussion section lacks other studies investigating CEUS of uterine fibroids as well as other treatment modalities (with much lower recurrence rates) such as uterine artery embolization (UAE).

Abstract
Please include the exceptionally good agreement indicated by an R2 value of 0.97.

**Thank you. This is mentioned in the Abstract; line 33.**

Introduction
There are non-thermal treatment options for uterine fibroids, such as UAE. These should at least be mentioned and some references provided.

**Thank you, we have previously published a randomized controlled trial comparing microwave ablation and UAE. We thus believe this is an important point! However, we have discussed this topic at length in that study (reference nr. 14) and to shorten the introduction and just report what we thought necessary for this study, we focused on MWA and more similar methods.** **If considered important, we would gladly expand on this topic and leave this to the discretion of the editor to decide.**

Methods
Line 86 – what made the subjects in this study “executive women”? Were they all CEOs or did the authors also include some mere VPs? Maybe the authors meant to say “consecutive”?

**Thank you for pointing out this obvious typo that we had missed. It has been changed!**

Please explain how the subjects in this study differed from the “more general population” included in the main study (line 90)?

**Thank you! This was unclear and has been changed to larger, which is a more appropriate description (line 90).**

Lines 104-105 – why was the number of subjects chosen to match a prior study? This is especially puzzling since the two studies are never compared to each other.

**This is an important point. As this was an exploratory study, no power calculation was performed for this outcome. We have clarified this in Methods; line 104-106.**

**The text now reads: The number of patients included in the sub-study was chosen to match a previous study from the same institution [14], as it was considered adequate for a feasibility study aimed at exploring the potential of CEUS for pre- and post-ablation assessment.**

Line 113 – GE Healthcare should be GE HealthCare.

**Thank you, it has been changed.**

Results
(Figure 1 does not show a comparison of pre- and post-ablation volumes within a week measured by contrast enhanced MRI and CEUS (lines 156-158). Please rewrite.

**Thank you for observing this, it has been changed.**

Why is the R2 value of 0.97 never mentioned in the main text (lines 177-178)?
I agree that an ICC over 0.75 indicates good agreement, but since the authors achieved an ICC of 0.96 (so > 0.90) this is excellent agreement.

**It is good point. However, as ICC reflects rank-order consistency it was mainly used to justify the Bland-Altman analysis.**

CEUS enabled immediate retreatment in 6 cases. (32%), which would seem a major advantage of using CEUS that should be discussed more (not necessarily in the Results section).

**Thank you for bringing this up! It is important and addressed in the Discussion; lines 239-243.**

**The text now reads: Using CEUS for immediate evaluation in a peri-operative setting enables visualization of any remaining vascularization within treated fibroids which can then be complimentary ablated in the same session. In our study, 6 fibroids out of 19 (32%) had an immediate additional ablation after the evaluation with CEUS. This must be considered a major advantage of CEUS over MRI.**

Discussion
This section lacks a comparison with other studies investigating CEUS of uterine fibroids (e.g., doi: 10.1007/s00261-026-05402-7, doi: 10.1016/j.ultrasmedbio.2023.11.012, doi: 10.1016/j.acra.2023.04.021, doi: 10.1002/jcu.23294)

**We thank you for the references, which we have evaluated. Although these references are about CEUS, we feel that our study focuses on comparison of evaluation of the ablation zone with CEUS vs MRI, and not merely on how CEUS can predict ablation effect. We also believe that our study shows the feasibility of the use of intra-operative CEUS for immediate evaluation of the ablation effects and the ability of CEUS to influence the treatment provided. Also see earlier response regarding other minimally invasive methods (that we have largely omitted in this study but addressed in earlier work).**

Considering that other treatment options have success rates above 95% (e.g., UAE) the fact that 47% of MWA treated fibroids recurred within 12 months would seem very important to discuss.

**Thank you for pointing this out. Our group has previously published a comparative trial of MWA and UAE including data on pain and post-operative sick leave etc. We have now included a mention of this in the discussion, line 319-323.**

**The text now reads: We propose that re-ablation could be an option also for uterine fibroids showing signs of regrowth or inadequate ablation results, especially since the patient acceptance of the method is high [14]. Treatment methods like uterine artery embolization (UAE) may be as effective [14] but not as well tolerated as MWA [24]. Our intention is to investigate the efficacy of reablation further in future studies.**

This study found no statistically (let alone clinically) significant differences between CEUS and MRI volume measurements, it seems odd to highlight the need for “adequate training” with CEUS (lines 241-243)

**Thank you for this comment. In our setting, gynaecologists are not usually trained in use of CEUS which is most often performed by radiologists. If CEUS will be used for evaluation of the ablation zone after ablation performed by gynaecologists, we do feel that adequate training of the use of CEUS is pertinent. We have modified the text in the discussion and hope that you agree.**

**The text now reads: If CEUS replaces MRI for evaluation of MWA treatment of uterine fibroids, adequate training of the provider in use of CEUS is necessary We used a commercially available product for CEUS and a well-established technique for fibroid evaluation. We therefore believe that our results are generalizable to any setting with corresponding experience with ultrasound evaluation with CEUS.**
