# Supplementary material for: Evaluation of the accuracy of peri-operative CEUS in measuring the effect of microwave ablation treatment of uterine fibroids, in comparison with MRI
Source: PLoS One. 2026 Aug 25;21(8):e0356141. doi: 10.1371/journal.pone.0356141 (PMC13505896; doi:10.1371/journal.pone.0356141)
Supplement: Supplementary file 2 [file pone.0356141.s003.docx]

**Response to Reviewers**

Thank you so much for taking the time to review the manuscript again. Below, our response is in **bold print.** The line references correspond to the Revised Manuscript with Track Changes. Note that references are not included in the text, for readability.

Reviewer #2: Generally speaking the authors have done a good job at answering my queries.

1. However, I still don't feel that having previously discussed other uterine fibroid treatment options in another paper justifies incomplete Introduction and Discussion sections. It is important to provide readers with more of an overview of the clinical options than is currently included.

**1. Thank you for this comment! We have now added a paragraph on other non- or minimally invasive methods in the Introduction (lines 49-71) which now reads:**

“In recent years, increasing attention has been paid to minimally invasive therapies such as high-intensity focused ultrasound (HIFU), uterine artery embolization (UAE), radiofrequency ablation (RFA) and percutaneous microwave ablation (MWA), with focus on uterine preservation and symptom improvement. HIFU is a totally non-invasive but time-consuming method, and best suited for women with fewer fibroids and smaller uteri. Although non-invasive, bowel or other organs in the way of the ultrasound beam constitutes a contraindication to treatment. UAE is an established method to decrease volume of fibroids and improve clinical symptoms, even though post-operative pain and post-embolization syndrome (PES) associated with the procedure have somewhat limited its use. There has also been concern that UAE may increase the risk of earlier menopause by affecting ovarian function, and the risk appears to be greater in women older than 40 years. However, previous studies have yielded conflicting results. RFA is a thermal ablation technique inducing coagulative necrosis of tissue around the tip of an antenna placed in the target of interest. For treatment of fibroids, RFA has mainly been used with a laparoscopic or, of late, vaginal approach even though there are studies of percutaneous RFA showing promising results. Like RFA, microwave ablation (MWA) generates heat and causes coagulative necrosis. Used correctly, it is safe, fast and easy to perform, and it is well tolerated by patients. Compared to the other non- or minimally invasive thermal techniques discussed above, MWA has the advantage of consistently higher tissue temperatures leading to larger ablation volumes in shorter time. MWA is also consistently effective independent of tissue types, leading to a more predictable outcome.”
**As the focus of this study is volume measurement with CEUS compared to MRI, we think further comparison of MWA with other techniques in the Discussion section is redundant.**

2. Similarly, there may not be other intra-operative CEUS studies of uterine fibroids, but clearly there have been other investigations of CEUS in this clinical space and that should be acknowledged and put in context with the current study.

**2. We have added to the discussion on this topic, by describing the conclusions of the Chinese studies previously just given as references. Lines 257-262, the text now reads:**

**“**Our results, as presented in Table 2, are in agreement with previous studies from China. In a study from 2014, Lei et al. found good correlation between ablation volumes measured by CEUS and post-operative contrast enhanced MRI, and Zhang et al. concluded that CEUS could even be advantageous over MRI as it gives reliable real-time information on the perfusion and possible viable fibroid residue within the ablated area. Our study confirms these results in a different setting adding to the generalizability of the method, which is of importance before implementing it in a clinical practice outside of Asia.”
**Also, there was already a comparison in the Discussion section between one of these studies (reference 19) and our study, lines 274-278.**

Reviewer #3:

1. Should the conclusion that CEUS can replace MRI be further softened, given the wide Bland–Altman limits and exploratory sample size?
**Thank you for your comment! However, we believe we have disclosed the limitations of the study in the Discussion section and been careful enough in the conclusion, with the change of the word “can” to “could” in the last paragraph (line 353).**

2. Could the 4-day median delay between CEUS and MRI have introduced systematic bias in ablation-volume estimates?
**Thank you for raising this question! We do not think so, as already discussed in the Discussion section line 291-301.** **However, in case of future similar studies, we will consider repeating the CEUS on the day of the post-operative MRI.**

3. Can the authors report 95% confidence intervals for the recurrence/regrowth rate and avoid overinterpreting subgroup findings? **Thank you for pointing this out. The confidence interval for the regrowth rate has been added. Lines 242-243 (95% CI: 26.2%–69.0%). It has also been added to the Discussion section (line 326) and the text has been changed to:** “The wide confidence interval due to the small sample size makes it difficult to generalize the results, but we proposed that when a rim of the fibroid must be left untreated due to the safety of surrounding structures as discussed above, there is a potential for regrowth.” **(Lines 329-332.)**
**We assume that subgroups apply to FIGO classification. We agree that overinterpreting should be avoided and have tried to address this in the paragraph reading:** “The mean ablation ratio was 90%, 71% and 78% respectively for FIGO groups 1-3 which was not a significant difference, but the sample size is too small for generalization.” **(Lines 184-186.)**

4. How might the lack of blinding and inter-/intra-observer reliability assessment affect the robustness of the CEUS–MRI comparison?
**Thank you for bringing this up. We agree it may need further clarification. The post-operative MRI was read without comparison with the CEUS measurements and the agreement between the methods was evaluated after all data had been collected, which should limit the effect of the lack of blinding. As stated in the Methods section lines 130-131, if needed there was the option of discussing the MRI with a second radiologist until a consensus agreement was reached and this was also true for CEUS. A paragraph about this has been added to the Discussion section lines 341-344: “**A limitation to the study is the lack of blinding and inter-/intra-observer reliability assessment of imaging (CEUS and MRI). However, as the volumes measured by CEUS and MRI were compared after all data were collected and there was an option of discussion among colleagues until consensus was reached, we believe the effect on the results is limited.”
