# Supplementary material for: Evaluation of the accuracy of peri-operative CEUS in measuring the effect of microwave ablation treatment of uterine fibroids, in comparison with MRI
Source: PLoS One. 2026 Aug 25;21(8):e0356141. doi: 10.1371/journal.pone.0356141 (PMC13505896; doi:10.1371/journal.pone.0356141)
Supplement: Peer review history

# Peer review: Decision Letter 0

**DOI:** 10.1371/journal.pone.0356141.r001

**Authors:** Yufeng Zhou

4 May 2026

PLOS One

Dear Dr. Beermann,

Thank you for submitting your manuscript to PLOS ONE. After careful consideration, we feel that it has merit but does not fully meet PLOS ONE’s publication criteria as it currently stands. Therefore, we invite you to submit a revised version of the manuscript that addresses the points raised during the review process.

Please submit your revised manuscript by Jun 18 2026 11:59PM. If you will need more time than this to complete your revisions, please reply to this message or contact the journal office at plosone@plos.org. When you're ready to submit your revision, log on to https://www.editorialmanager.com/pone/ and select the 'Submissions Needing Revision' folder to locate your manuscript file.

- A letter that responds to each point raised by the academic editor and reviewer(s). You should upload this letter as a separate file labeled 'Response to Reviewers'.
- A marked-up copy of your manuscript that highlights changes made to the original version. You should upload this as a separate file labeled 'Revised Manuscript with Track Changes'.
- An unmarked version of your revised paper without tracked changes. You should upload this as a separate file labeled 'Manuscript'.

If you would like to make changes to your financial disclosure, please include your updated statement in your cover letter. Guidelines for resubmitting your figure files are available below the reviewer comments at the end of this letter.

If applicable, we recommend that you deposit your laboratory protocols in protocols.io to enhance the reproducibility of your results. Protocols.io assigns your protocol its own identifier (DOI) so that it can be cited independently in the future. For instructions see: https://journals.plos.org/plosone/s/submission-guidelines#loc-laboratory-protocols. Additionally, PLOS ONE offers an option for publishing peer-reviewed Lab Protocol articles, which describe protocols hosted on protocols.io. Read more information on sharing protocols at https://plos.org/protocols?utm_medium=editorial-email&utm_source=authorletters&utm_campaign=protocols.

As the corresponding author, your ORCID iD is verified in the submission system and will appear in the published article. PLOS supports the use of ORCID, and we encourage all coauthors to register for an ORCID iD and use it as well. Please encourage your coauthors to verify their ORCID iD within the submission system before final acceptance, as unverified ORCID iDs will not appear in the published article. *Only*  the individual author can complete the verification step; PLOS staff *cannot*  verify ORCID iDs on behalf of authors.

We look forward to receiving your revised manuscript.

Kind regards,

Yufeng Zhou, PhD

Academic Editor

PLOS One

**Journal Requirements:**

When submitting your revision, we need you to address these additional requirements.

1. Please ensure that your manuscript meets PLOS ONE's style requirements, including those for file naming. The PLOS ONE style templates can be found at

https://journals.plos.org/plosone/s/file?id=wjVg/PLOSOne_formatting_sample_main_body.pdf and

https://journals.plos.org/plosone/s/file?id=ba62/PLOSOne_formatting_sample_title_authors_affiliations.pdf

2. We note that the grant information you provided in the ‘Funding Information’ and ‘Financial Disclosure’ sections do not match.

When you resubmit, please ensure that you provide the correct grant numbers for the awards you received for your study in the ‘Funding Information’ section.

3. Please note that funding information should not appear in any section or other areas of your manuscript. We will only publish funding information present in the Funding Statement section of the online submission form. Please remove any funding-related text from the manuscript.

4. Thank you for stating the following in the Competing Interests section:

“Marie Beermann reports a relationship with Medtronic that includes non-financial support and travel reimbursement.

Helena Kopp-Kallner reports a relationship with Medtronic that includes non-financial support and travel reimbursement. Helena Kopp-Kallner reports a relationship with Mermaid Medical that includes non-financial support.

Other authors declare that they have no known competing financial interests or personal relationships that could have appeared to influence the work reported in this paper.”

Please confirm that this does not alter your adherence to all PLOS ONE policies on sharing data and materials, by including the following statement: "This does not alter our adherence to  PLOS ONE policies on sharing data and materials.” (as detailed online in our guide for authors http://journals.plos.org/plosone/s/competing-interests). If there are restrictions on sharing of data and/or materials, please state these. Please note that we cannot proceed with consideration of your article until this information has been declared.

Please respond by return email with your amended Competing Interests Statement and we will change the online submission form on your behalf.

5. Please provide a complete Data Availability Statement in the submission form, ensuring you include all necessary access information or a reason for why you are unable to make your data freely accessible. If your research concerns only data provided within your submission, please write "All data are in the manuscript and/or supporting information files" as your Data Availability Statement.

6. If the reviewer comments include a recommendation to cite specific previously published works, please review and evaluate these publications to determine whether they are relevant and should be cited. There is no requirement to cite these works unless the editor has indicated otherwise.

[Note: HTML markup is below. Please do not edit.]

Reviewers' comments:

Reviewer's Responses to Questions

**Comments to the Author**

1. Is the manuscript technically sound, and do the data support the conclusions?

Reviewer #1: No

Reviewer #2: Yes

**********

2. Has the statistical analysis been performed appropriately and rigorously?

Reviewer #1: No

Reviewer #2: Yes

**********

3. Have the authors made all data underlying the findings in their manuscript fully available?

The PLOS Data policy

Reviewer #1: No

Reviewer #2: Yes

**********

4. Is the manuscript presented in an intelligible fashion and written in standard English?

Reviewer #1: No

Reviewer #2: Yes

**********

Reviewer #1: This study addresses a clinically meaningful question — whether intraoperative CEUS can replace MRI for evaluating microwave ablation of uterine fibroids — and the methodological approach is reasonable in its general design. However, the manuscript has several fundamental limitations that prevent the data from supporting the conclusions as currently stated.

1. Interchangeability is not established

The primary conclusion that CEUS and MRI "can be used interchangeably" is not justified by the data. The Bland-Altman limits of agreement span 88.6 ml (−45.98 to 42.61 ml), representing approximately 85% of the mean ablated volume. No minimum clinically important difference (MCID) was prespecified, making it impossible to determine whether this range of disagreement is clinically acceptable. The authors are reminded that a high ICC reflects rank-order consistency, not method interchangeability; the two must not be conflated.

2. Timing bias is inadequately addressed

CEUS was performed intraoperatively, while MRI was obtained within one week of ablation. Post-ablation inflammatory edema may systematically expand the non-enhancing zone visible on MRI, introducing a directional bias into the volume comparison. The authors briefly mention acoustic bubble interference as a limitation of CEUS but do not analyze or quantify the potential effect of this timing difference. The actual median interval between procedures should be reported, and its implications for the results must be discussed.

3. No sample size justification

No a priori sample size calculation is provided for either the primary or secondary outcomes. Matching the sample size of a prior study from the same institution does not constitute a methodological justification. The authors should either provide a formal power calculation or explicitly frame this as a feasibility study, with conclusions worded accordingly.4. Secondary outcomes are statistically underpowered

The recurrence rate of 47% is based on 8 events among 15 patients with complete follow-up. The corresponding 95% confidence interval spans approximately 21% to 73% — far too wide to be clinically informative — yet no confidence interval is reported in the manuscript. The subgroup analysis by FIGO classification includes only 2 fibroids in the subserosal group; reporting a non-significant difference across groups at this sample size risks being misinterpreted as evidence of equivalence.

5. Ablation ratio data are internally inconsistent

The text states that ablation volume exceeded fibroid volume in two cases. Table 2 shows ablation ratios exceeding 100% in more than two cases (e.g., Patient 2 CEUS 123%, Patient 16 MRI 157%). This discrepancy must be fully reconciled, with case-by-case explanation provided for all instances where the ratio exceeds 100%.6. Additional concerns

The recurrence outcome ("regrowth of vascularized tissue") lacks an operational definition. Whether this refers to new contrast enhancement within the ablation zone or an increase in overall fibroid volume should be specified, as the distinction carries different clinical and pathophysiological implications.

The CEUS volume calculation method is never described. MRI uses the ellipsoid formula (length × width × height × 0.52); it is unclear whether the same formula was applied to CEUS measurements.

Neither blinding nor inter- or intra-observer reliability is reported for either modality. Given that MRI serves as the reference standard, its measurement reproducibility should be established.

The ICC model specification (one-way vs. two-way; absolute agreement vs. consistency; single vs. average measures) is not reported and must be added.

One data point in Figure 3 visually exceeds the upper limit of agreement but is not identified or discussed in the text. This outlier should be labeled and addressed.

Reviewer #2: Review of PONE-D-26-16095

Evaluation of the accuracy of peri-operative CEUS in measuring the effect of microwave ablation treatment of uterine fibroids, in comparison with MRI

Summary and general comments

The authors present results from a small study in 19 women using CEUS and MRI to measure ablation volumes and outcomes after MWA of uterine fibroids. The authors found an R2 value of 0.97 between CEUS and MRI volumes. CEUS enabled immediate retreatment in 6 cases. Overall 47% of the fibroids showed recurrence after one year. The is an interesting manuscript, but the excellent agreement between CEUS and MRI is somewhat understated and the Discussion section lacks other studies investigating CEUS of uterine fibroids as well as other treatment modalities (with much lower recurrence rates) such as uterine artery embolization (UAE).

Abstract

Please include the exceptionally good agreement indicated by an R2 value of 0.97.

Introduction

There are non-thermal treatment options for uterine fibroids, such as UAE. These should at least be mentioned and some references provided.

Methods

Line 86 – what made the subjects in this study “executive women”? Were they all CEOs or did the authors also include some mere VPs? Maybe the authors meant to say “consecutive”?

Please explain how the subjects in this study differed from the “more general population” included in the main study (line 90)?

Lines 104-105 – why was the number of subjects chosen to match a prior study? This is especially puzzling since the two studies are never compared to each other.

Line 113 – GE Healthcare should be GE HealthCare.

Results

(Figure 1 does not show a comparison of pre- and post-ablation volumes within a week measured by contrast enhanced MRI and CEUS (lines 156-158). Please rewrite.

Why is the R2 value of 0.97 never mentioned in the main text (lines 177-178)?

I agree that an ICC over 0.75 indicates good agreement, but since the authors achieved an ICC of 0.96 (so > 0.90) this is excellent agreement.

CEUS enabled immediate retreatment in 6 cases. (32%), which would seem a major advantage of using CEUS that should be discussed more (not necessarily in the Results section).

Discussion

This section lacks a comparison with other studies investigating CEUS of uterine fibroids (e.g., doi: 10.1007/s00261-026-05402-7, doi: 10.1016/j.ultrasmedbio.2023.11.012, doi: 10.1016/j.acra.2023.04.021, doi: 10.1002/jcu.23294)

Considering that other treatment options have success rates above 95% (e.g., UAE) the fact that 47% of MWA treated fibroids recurred within 12 months would seem very important to discuss.

This study found no statistically (let alone clinically) significant differences between CEUS and MRI volume measurements, it seems odd to highlight the need for “adequate training” with CEUS (lines 241-243)

**********

what does this mean?). If published, this will include your full peer review and any attached files.

If you choose “no”, your identity will remain anonymous but your review may still be made public.

**Do you want your identity to be public for this peer review?**  For information about this choice, including consent withdrawal, please see our Privacy Policy

Reviewer #1: No

Reviewer #2: No

**********

[NOTE: If reviewer comments were submitted as an attachment file, they will be attached to this email and accessible via the submission site. Please log into your account, locate the manuscript record, and check for the action link "View Attachments". If this link does not appear, there are no attachment files.]

To ensure your figures meet our technical requirements, please review our figure guidelines: https://journals.plos.org/plosone/s/figures

You may also use PLOS’s free figure tool, NAAS, to help you prepare publication quality figures: https://journals.plos.org/plosone/s/figures#loc-tools-for-figure-preparation.

NAAS will assess whether your figures meet our technical requirements by comparing each figure against our figure specifications.

# Author comment: Author response to Decision Letter 1

**DOI:** 10.1371/journal.pone.0356141.r002

4 Jun 2026

Response to Reviewers and the Editor

Thank you so much for taking the time to review this manuscript. We appreciate the chance to improve our work according to your remarks! The line references correspond to the Revised Manuscript with Track Changes.

Journal Requirements:

1. Please ensure that your manuscript meets PLOS ONE's style requirements, including those for file naming.

Thank you, we have tried to change the manuscript files according to your requirements.

Note that Fig. 1 constitutes two images saved in the required format as Picture1a and Picture1b. The complete figure, as created by the author, is also included as a template.

2. We note that the grant information you provided in the ‘Funding Information’ and ‘Financial Disclosure’ sections do not match.

When you resubmit, please ensure that you provide the correct grant numbers for the awards you received for your study in the ‘Funding Information’ section.

Checked and changed.

3. Please note that funding information should not appear in any section or other areas of your manuscript. We will only publish funding information present in the Funding Statement section of the online submission form. Please remove any funding-related text from the manuscript.

Thank you, this information has been removed from the manuscript.

4. Thank you for stating the following in the Competing Interests section:

“Marie Beermann reports a relationship with Medtronic that includes non-financial support and travel reimbursement.

Helena Kopp Kallner reports a relationship with Medtronic that includes non-financial support and travel reimbursement. Helena Kopp-Kallner reports a relationship with Mermaid Medical that includes non-financial support.

Other authors declare that they have no known competing financial interests or personal relationships that could have appeared to influence the work reported in this paper.”

Please confirm that this does not alter your adherence to all PLOS ONE policies on sharing data and materials, by including the following statement: "This does not alter our adherence to PLOS ONE policies on sharing data and materials.” (as detailed online in our guide for authors http://journals.plos.org/plosone/s/competing-interests). If there are restrictions on sharing of data and/or materials, please state these. Please note that we cannot proceed with consideration of your article until this information has been declared.

Please respond by return email with your amended Competing Interests Statement and we will change the online submission form on your behalf.

Thank you! It has been done.

5. Please provide a complete Data Availability Statement in the submission form, ensuring you include all necessary access information or a reason for why you are unable to make your data freely accessible. If your research concerns only data provided within your submission, please write "All data are in the manuscript and/or supporting information files" as your Data Availability Statement.

Thank you, we are providing this data availability statement:

Data is provided as in the manuscript. More detailed data including the full dataset can be shared within collaborative efforts after signing agreements for data sharing and/or transfer. Data containing personal information cannot be shared without agreement, according to the General Data Protection Regulation (GDPR).

6. If the reviewer comments include a recommendation to cite specific previously published works, please review and evaluate these publications to determine whether they are relevant and should be cited. There is no requirement to cite these works unless the editor has indicated otherwise.

Not applicable.

Reviewers' comments:

Reviewer #1: This study addresses a clinically meaningful question — whether intraoperative CEUS can replace MRI for evaluating microwave ablation of uterine fibroids — and the methodological approach is reasonable in its general design. However, the manuscript has several fundamental limitations that prevent the data from supporting the conclusions as currently stated.

1. Interchangeability is not established

The primary conclusion that CEUS and MRI "can be used interchangeably" is not justified by the data. The Bland-Altman limits of agreement span 88.6 ml (−45.98 to 42.61 ml), representing approximately 85% of the mean ablated volume. No minimum clinically important difference (MCID) was prespecified, making it impossible to determine whether this range of disagreement is clinically acceptable. The authors are reminded that a high ICC reflects rank-order consistency, not method interchangeability; the two must not be conflated.

We thank you for this meaningful comment. This has been changed and/or addressed in the Abstract (line 38-39), the Introduction (line 78-79) and the Discussion (327-329) and the word interchangeable is no longer used in the manuscript.

The text now reads:

Abstract: CEUS can be used to evaluate ablation volume and ablation ratio and is useful for intraoperative estimation and guidance. It shows strong correlation with MRI.

Introduction: Accuracy and generalizability of peri-operative CEUS has been established [12] but the aim of this study was to reproduce the results and to investigate if it can be used for accurate estimation of the ablation volume in a clinical setting where MRI is not available.

Discussion: Also, even with the current sample size, the correlation between CEUS and MRI was strong enough to support CEUS for intraoperative estimation and guidance.

2. Timing bias is inadequately addressed

CEUS was performed intraoperatively, while MRI was obtained within one week of ablation. Post-ablation inflammatory edema may systematically expand the non-enhancing zone visible on MRI, introducing a directional bias into the volume comparison. The authors briefly mention acoustic bubble interference as a limitation of CEUS but do not analyze or quantify the potential effect of this timing difference. The actual median interval between procedures should be reported, and its implications for the results must be discussed.

We agree that information about timing of the post-operative MRI is missing and have added the median interval between ablation and MRI (Results; line 161-162) and the implication of this timing is further discussed (Discussion; line 274-285).

The text now reads:

Results: The mean time between ablation and post-operative MRI was 5,5 days, median 4 days.

Discussion: For logistical reasons, the post-operative MRI could not be performed immediately following the ablation. The mean time between ablation and post-operative MRI was 5,5 days (median 4 days). A study of microwave ablation of liver tumors by Alzubaidi et al. [19] found that the ablation zone expanded during the first 24 hours after treatment, likely due to evolving edema and tissue response. None of our patients had the post-operative MRI performed within the first 48 hours. Thus, remaining post-operative expansion of the non-enhancing zone is not likely to affect results but as discussed previously, large lesions are difficult to evaluate with ultrasound. For most cases (68%), the absolute difference between volume measured by CEUS and MRI was less than 10 ml and in conclusion we argue that the CEUS evaluation can be performed intra-operatively and that the two methods agree sufficiently to support the use of CEUS in our clinical setting of outpatient treatment.

However, we do not agree that the ablation zone will continually expand after 24 h, and as none of the patients did an MRI within 48 h we do not believe that the timing is the reason for the differences in volume (but rather the difficulty of measuring large lesions with ultrasound, as discussed).

3. No sample size justification

No a priori sample size calculation is provided for either the primary or secondary outcomes. Matching the sample size of a prior study from the same institution does not constitute a methodological justification. The authors should either provide a formal power calculation or explicitly frame this as a feasibility study, with conclusions worded accordingly.

We agree. As this was an exploratory study, no power calculation was performed for this outcome. We have clarified this in Methods; line 104-106.

The text now reads: The number of patients included in the sub-study was chosen to match a previous study from the same institution [14], as it was considered adequate for a feasibility study aimed at exploring the potential of CEUS for pre- and post-ablation assessment.

4. Secondary outcomes are statistically underpowered

The recurrence rate of 47% is based on 8 events among 15 patients with complete follow-up. The corresponding 95% confidence interval spans approximately 21% to 73% — far too wide to be clinically informative — yet no confidence interval is reported in the manuscript. The subgroup analysis by FIGO classification includes only 2 fibroids in the subserosal group; reporting a non-significant difference across groups at this sample size risks being misinterpreted as evidence of equivalence.

Thank you for pointing this out. However, we do feel that it is clinically important to point out recurrences, as these are seldomly transparently reported in the literature. We have added a line addressing this (Results; line 178) and deleted the lines 228-230 (... no signs of regrowth were seen in the 2 subserosal fibroids included in the study.)

Added line: ...but the sample size is too small for generalization.

5. Ablation ratio data are internally inconsistent

The text states that ablation volume exceeded fibroid volume in two cases. Table 2 shows ablation ratios exceeding 100% in more than two cases (e.g., Patient 2 CEUS 123%, Patient 16 MRI 157%). This discrepancy must be fully reconciled, with case-by-case explanation provided for all instances where the ratio exceeds 100%.

Thank you for bringing this up as it needed clarification. It is always difficult to tell exactly where the original tumor is localized within an ablation zone, hence we chose to measure the whole avascular area. This is further discussed in Discussion; line 299-307.

The text now reads: In ablation of malignant liver tumors, minimal ablative margin (MAM) is important for improving local control following thermal ablation[20]. At least 5 mm of healthy tissue surrounding the tumor should be covered by the ablation zone, to reduce the risk of local recurrence. When it comes to benign lesions like fibroids safety comes first, but if there are no heat sensitive structures at risk the aim should be complete ablation. For four fibroids the ablation ratio exceeded 100%. All were classified as FIGO group 1 (submucosal) which, due to location, allowed more aggressive treatment and thus an ablation zone covering more than the actual lesion. More commonly in this study, the outermost rim of the fibroid was left to avoid harm.

6. Additional concerns

The recurrence outcome ("regrowth of vascularized tissue") lacks an operational definition. Whether this refers to new contrast enhancement within the ablation zone or an increase in overall fibroid volume should be specified, as the distinction carries different clinical and pathophysiological implications.

Thank you! We have added this under Results; line 227-228 and Discussion; line 311-315.

The text now reads:

Results: Regrowth of vascularized fibroid tissue, defined as recurrent contrast enhancement in treated lesions, was seen in 8 fibroids (47%).

Discussion: Also, in almost 50% of ablated patients there was regrowth of vascularized fibroid tissue defined as recurrent contrast enhancement in treated lesions, even though the ablation volume (i.e. the avascular part of the fibroid) continued to shrink. When a rim of the fibroid must be left untreated due to the safety of surrounding structures as discussed above, there is a potential for regrowth.

The CEUS volume calculation method is never described. MRI uses the ellipsoid formula (length × width × height × 0.52); it is unclear whether the same formula was applied to CEUS measurements.

We apologize. It has been added under Methods; line 127 (width x height x length x 0.52).

Neither blinding nor inter- or intra-observer reliability is reported for either modality. Given that MRI serves as the reference standard, its measurement reproducibility should be established.

Thank you for addressing this, which is clearly a weakness of our study. We have touched on this under Methods; line 123 (“discussed with a second radiologist until a consensus agreement was reached”). We did not have the resources to do a proper inter-observer reliability study.

The ICC model specification (one-way vs. two-way; absolute agreement vs. consistency; single vs. average measures) is not reported and must be added.

One data point in Figure 3 visually exceeds the upper limit of agreement but is not identified or discussed in the text. This outlier should be labeled and addressed.

A clarification of the ICC model has been added to Statistical analysis; line 148-149. It should be one-way, absolute agreement.

It is true that there was one clear outlier. This text was included in the results section: The graph shows that all points but one fall within the 95% limit of agreement (line 218-219). We have added information on this in the discussion.

The text now reads: The largest difference between the ablation volume measured by CEUS and MRI in this study was 63 ml in a clear outlier. It was noted in the second largest fibroid with a pre-operative volume of 298 ml (MRI). For large lesions, ultrasound may have the limitation of smaller field of view than MRI. In addition, a characteristic of fibroids is echo-shadowing, which further complicates the measurement of large fibroids and for these lesions MRI is a more precise method Due to technical reasons it is also difficult to achieve spherical ablation zones larger than 5-6 cm in diameter [18], and for very large fibroids other treatments can be considered as more appropriate.

Reviewer #2: Review of PONE-D-26-16095

Evaluation of the accuracy of peri-operative CEUS in measuring the effect of microwave ablation treatment of uterine fibroids, in comparison with MRI

Summary and general comments

The authors present results from a small study in 19 women using CEUS and MRI to measure ablation volumes and outcomes after MWA of uterine fibroids. The authors found an R2 value of 0.97 between CEUS and MRI volumes. CEUS enabled immediate retreatment in 6 cases. Overall 47% of the fibroids showed recurrence after one year. The is an interesting manuscript, but the excellent agreement between CEUS and MRI is somewhat understated and the Discussion section lacks other studies investigating CEUS of uterine fibroids as well as other treatment modalities (with much lower recurrence rates) such as uterine artery embolization (UAE).

Abstract

Please include the exceptionally good agreement indicated by an R2 value of 0.97.

Thank you. This is mentioned in the Abstract; line 33.

Introduction

There are non-thermal treatment options for uterine fibroids, such as UAE. These should at least be mentioned and some references provided.

Thank you, we have previously published a randomized controlled trial comparing microwave ablation and UAE. We thus believe this is an important point! However, we have discussed this topic at length in that study (reference nr. 14) and to shorten the introduction and just report what we thought necessary for this study, we focused on MWA and more similar methods. If considered important, we would gladly expand on this topic and leave this to the discretion of the editor to decide.

Methods

Line 86 – what made the subjects in this study “executive women”? Were they all CEOs or did the authors also include some mere VPs? Maybe the authors meant to say “consecutive”?

Thank you for pointing out this obvious typo that we had missed. It has been changed!

Please explain how the subjects in this study differed from the “more general population” included in the main study (line 90)?

Thank you! This was unclear and has been changed to larger, which is a more appropriate description (line 90).

Lines 104-105 – why was the number of sub

Attachment Submitted filename: Response to Reviewers3.docx

# Peer review: Decision Letter 1

**DOI:** 10.1371/journal.pone.0356141.r003

**Authors:** Yufeng Zhou

8 Jul 2026

Dear Dr. Beermann,

Thank you for submitting your manuscript to PLOS ONE. After careful consideration, we feel that it has merit but does not fully meet PLOS ONE’s publication criteria as it currently stands. Therefore, we invite you to submit a revised version of the manuscript that addresses the points raised during the review process.

Please submit your revised manuscript by Aug 22 2026 11:59PM. If you will need more time than this to complete your revisions, please reply to this message or contact the journal office at plosone@plos.org. When you're ready to submit your revision, log on to https://www.editorialmanager.com/pone/ and select the 'Submissions Needing Revision' folder to locate your manuscript file.

- A letter that responds to each point raised by the academic editor and reviewer(s). You should upload this letter as a separate file labeled 'Response to Reviewers'.
- A marked-up copy of your manuscript that highlights changes made to the original version. You should upload this as a separate file labeled 'Revised Manuscript with Track Changes'.
- An unmarked version of your revised paper without tracked changes. You should upload this as a separate file labeled 'Manuscript'.

If you would like to make changes to your financial disclosure, please include your updated statement in your cover letter. Guidelines for resubmitting your figure files are available below the reviewer comments at the end of this letter.

If applicable, we recommend that you deposit your laboratory protocols in protocols.io to enhance the reproducibility of your results. Protocols.io assigns your protocol its own identifier (DOI) so that it can be cited independently in the future. For instructions see: https://journals.plos.org/plosone/s/submission-guidelines#loc-laboratory-protocols. Additionally, PLOS ONE offers an option for publishing peer-reviewed Lab Protocol articles, which describe protocols hosted on protocols.io. Read more information on sharing protocols at https://plos.org/protocols?utm_medium=editorial-email&utm_source=authorletters&utm_campaign=protocols.

As the corresponding author, your ORCID iD is verified in the submission system and will appear in the published article. PLOS supports the use of ORCID, and we encourage all coauthors to register for an ORCID iD and use it as well. Please encourage your coauthors to verify their ORCID iD within the submission system before final acceptance, as unverified ORCID iDs will not appear in the published article. *Only*  the individual author can complete the verification step; PLOS staff *cannot*  verify ORCID iDs on behalf of authors.

We look forward to receiving your revised manuscript.

Kind regards,

Yufeng Zhou, PhD

Academic Editor

PLOS One

Journal Requirements:

If the reviewer comments include a recommendation to cite specific previously published works, please review and evaluate these publications to determine whether they are relevant and should be cited. There is no requirement to cite these works unless the editor has indicated otherwise.

Please review your reference list to ensure that it is complete and correct. If you have cited papers that have been retracted, please include the rationale for doing so in the manuscript text, or remove these references and replace them with relevant current references. Any changes to the reference list should be mentioned in the rebuttal letter that accompanies your revised manuscript. If you need to cite a retracted article, indicate the article’s retracted status in the References list and also include a citation and full reference for the retraction notice.

[Note: HTML markup is below. Please do not edit.]

Reviewers' comments:

Reviewer's Responses to Questions

**Comments to the Author**

Reviewer #2: (No Response)

Reviewer #3: All comments have been addressed

**********

2. Is the manuscript technically sound, and do the data support the conclusions?

Reviewer #2: Yes

Reviewer #3: Yes

**********

3. Has the statistical analysis been performed appropriately and rigorously?

Reviewer #2: Yes

Reviewer #3: No

**********

4. Have the authors made all data underlying the findings in their manuscript fully available?

The PLOS Data policy

Reviewer #2: Yes

Reviewer #3: No

**********

5. Is the manuscript presented in an intelligible fashion and written in standard English?

Reviewer #2: Yes

Reviewer #3: Yes

**********

Reviewer #2: Generally speaking the authors have done a good job at answering my queries. However, I still don't feel that having previously discussed other uterine fibroid treatment options in another paper justifies incomplete Introduction and Discussion sections. It is important to provide readers with more of an overview of the clinical options than is currently included. Similarly, there may not be other intra-operative CEUS studies of uterine fibroids, but clearly there have been other investigations of CEUS in this clinical space and that should be acknowledged and put in context with the current study.

Reviewer #3: 1. Should the conclusion that CEUS can replace MRI be further softened, given the wide Bland–Altman limits and exploratory sample size?

2. Could the 4-day median delay between CEUS and MRI have introduced systematic bias in ablation-volume estimates?

3. Can the authors report 95% confidence intervals for the recurrence/regrowth rate and avoid overinterpreting subgroup findings?

4. How might the lack of blinding and inter-/intra-observer reliability assessment affect the robustness of the CEUS–MRI comparison?

**********

what does this mean?). If published, this will include your full peer review and any attached files.

If you choose “no”, your identity will remain anonymous but your review may still be made public.

**Do you want your identity to be public for this peer review?**  For information about this choice, including consent withdrawal, please see our Privacy Policy

Reviewer #2: No

Reviewer #3: No

**********

[NOTE: If reviewer comments were submitted as an attachment file, they will be attached to this email and accessible via the submission site. Please log into your account, locate the manuscript record, and check for the action link "View Attachments". If this link does not appear, there are no attachment files.]

To ensure your figures meet our technical requirements, please review our figure guidelines: https://journals.plos.org/plosone/s/figures

You may also use PLOS’s free figure tool, NAAS, to help you prepare publication quality figures: https://journals.plos.org/plosone/s/figures#loc-tools-for-figure-preparation.

NAAS will assess whether your figures meet our technical requirements by comparing each figure against our figure specifications.

# Author comment: Author response to Decision Letter 2

**DOI:** 10.1371/journal.pone.0356141.r004

20 Jul 2026

Response to Reviewers

Thank you so much for taking the time to review the manuscript again. Below, our response is in bold print. The line references correspond to the Revised Manuscript with Track Changes. Note that references are not included in the text, for readability.

Reviewer #2: Generally speaking the authors have done a good job at answering my queries.

1. However, I still don't feel that having previously discussed other uterine fibroid treatment options in another paper justifies incomplete Introduction and Discussion sections. It is important to provide readers with more of an overview of the clinical options than is currently included.

1. Thank you for this comment! We have now added a paragraph on other non- or minimally invasive methods in the Introduction (lines 49-71) which now reads:

“In recent years, increasing attention has been paid to minimally invasive therapies such as high-intensity focused ultrasound (HIFU), uterine artery embolization (UAE), radiofrequency ablation (RFA) and percutaneous microwave ablation (MWA), with focus on uterine preservation and symptom improvement. HIFU is a totally non-invasive but time-consuming method, and best suited for women with fewer fibroids and smaller uteri. Although non-invasive, bowel or other organs in the way of the ultrasound beam constitutes a contraindication to treatment. UAE is an established method to decrease volume of fibroids and improve clinical symptoms, even though post-operative pain and post-embolization syndrome (PES) associated with the procedure have somewhat limited its use. There has also been concern that UAE may increase the risk of earlier menopause by affecting ovarian function, and the risk appears to be greater in women older than 40 years. However, previous studies have yielded conflicting results. RFA is a thermal ablation technique inducing coagulative necrosis of tissue around the tip of an antenna placed in the target of interest. For treatment of fibroids, RFA has mainly been used with a laparoscopic or, of late, vaginal approach even though there are studies of percutaneous RFA showing promising results. Like RFA, microwave ablation (MWA) generates heat and causes coagulative necrosis. Used correctly, it is safe, fast and easy to perform, and it is well tolerated by patients. Compared to the other non- or minimally invasive thermal techniques discussed above, MWA has the advantage of consistently higher tissue temperatures leading to larger ablation volumes in shorter time. MWA is also consistently effective independent of tissue types, leading to a more predictable outcome.”

As the focus of this study is volume measurement with CEUS compared to MRI, we think further comparison of MWA with other techniques in the Discussion section is redundant.

2. Similarly, there may not be other intra-operative CEUS studies of uterine fibroids, but clearly there have been other investigations of CEUS in this clinical space and that should be acknowledged and put in context with the current study.

2. We have added to the discussion on this topic, by describing the conclusions of the Chinese studies previously just given as references. Lines 257-262, the text now reads:

“Our results, as presented in Table 2, are in agreement with previous studies from China. In a study from 2014, Lei et al. found good correlation between ablation volumes measured by CEUS and post-operative contrast enhanced MRI, and Zhang et al. concluded that CEUS could even be advantageous over MRI as it gives reliable real-time information on the perfusion and possible viable fibroid residue within the ablated area. Our study confirms these results in a different setting adding to the generalizability of the method, which is of importance before implementing it in a clinical practice outside of Asia.”

Also, there was already a comparison in the Discussion section between one of these studies (reference 19) and our study, lines 274-278.

Reviewer #3:

1. Should the conclusion that CEUS can replace MRI be further softened, given the wide Bland–Altman limits and exploratory sample size?

Thank you for your comment! However, we believe we have disclosed the limitations of the study in the Discussion section and been careful enough in the conclusion, with the change of the word “can” to “could” in the last paragraph (line 353).

2. Could the 4-day median delay between CEUS and MRI have introduced systematic bias in ablation-volume estimates?

Thank you for raising this question! We do not think so, as already discussed in the Discussion section line 291-301. However, in case of future similar studies, we will consider repeating the CEUS on the day of the post-operative MRI.

3. Can the authors report 95% confidence intervals for the recurrence/regrowth rate and avoid overinterpreting subgroup findings? Thank you for pointing this out. The confidence interval for the regrowth rate has been added. Lines 242-243 (95% CI: 26.2%–69.0%). It has also been added to the Discussion section (line 326) and the text has been changed to: “The wide confidence interval due to the small sample size makes it difficult to generalize the results, but we proposed that when a rim of the fibroid must be left untreated due to the safety of surrounding structures as discussed above, there is a potential for regrowth.” (Lines 329-332.)

We assume that subgroups apply to FIGO classification. We agree that overinterpreting should be avoided and have tried to address this in the paragraph reading: “The mean ablation ratio was 90%, 71% and 78% respectively for FIGO groups 1-3 which was not a significant difference, but the sample size is too small for generalization.” (Lines 184-186.)

4. How might the lack of blinding and inter-/intra-observer reliability assessment affect the robustness of the CEUS–MRI comparison?

Thank you for bringing this up. We agree it may need further clarification. The post-operative MRI was read without comparison with the CEUS measurements and the agreement between the methods was evaluated after all data had been collected, which should limit the effect of the lack of blinding. As stated in the Methods section lines 130-131, if needed there was the option of discussing the MRI with a second radiologist until a consensus agreement was reached and this was also true for CEUS. A paragraph about this has been added to the Discussion section lines 341-344: “A limitation to the study is the lack of blinding and inter-/intra-observer reliability assessment of imaging (CEUS and MRI). However, as the volumes measured by CEUS and MRI were compared after all data were collected and there was an option of discussion among colleagues until consensus was reached, we believe the effect on the results is limited.”

Attachment Submitted filename: New Response to Reviewers.docx

# Peer review: Decision Letter 2

**DOI:** 10.1371/journal.pone.0356141.r005

**Authors:** Yufeng Zhou

30 Jul 2026

Evaluation of the accuracy of peri-operative CEUS in measuring the effect of microwave ablation treatment of uterine fibroids, in comparison with MRI

PONE-D-26-16095R2

Dear Dr. Beermann,

We’re pleased to inform you that your manuscript has been judged scientifically suitable for publication and will be formally accepted for publication once it meets all outstanding technical requirements.

Within one week, you’ll receive an e-mail detailing the required amendments. When these have been addressed, you’ll receive a formal acceptance letter and your manuscript will be scheduled for publication.

An invoice will be generated when your article is formally accepted. Please note, if your institution has a publishing partnership with PLOS and your article meets the relevant criteria, all or part of your publication costs will be covered. Please make sure your user information is up-to-date by logging into Editorial Manager at Editorial Manager® and clicking the ‘Update My Information' link at the top of the page. For questions related to billing, please contact billing support.

If your institution or institutions have a press office, please notify them about your upcoming paper to help maximize its impact. If they’ll be preparing press materials, please inform our press team as soon as possible -- no later than 48 hours after receiving the formal acceptance. Your manuscript will remain under strict press embargo until 2 pm Eastern Time on the date of publication. For more information, please contact onepress@plos.org.

Kind regards,

Yufeng Zhou, PhD

Academic Editor

PLOS One

Additional Editor Comments (optional):

Reviewers' comments:

Reviewer's Responses to Questions

**Comments to the Author**

Reviewer #2: All comments have been addressed

**********

2. Is the manuscript technically sound, and do the data support the conclusions?

Reviewer #2: (No Response)

**********

3. Has the statistical analysis been performed appropriately and rigorously?

Reviewer #2: (No Response)

**********

4. Have the authors made all data underlying the findings in their manuscript fully available?

The PLOS Data policy

Reviewer #2: (No Response)

**********

5. Is the manuscript presented in an intelligible fashion and written in standard English?

Reviewer #2: (No Response)

**********

Reviewer #2: (No Response)

**********

what does this mean?). If published, this will include your full peer review and any attached files.

If you choose “no”, your identity will remain anonymous but your review may still be made public.

**Do you want your identity to be public for this peer review?**  For information about this choice, including consent withdrawal, please see our Privacy Policy

Reviewer #2: No

**********

# Editor report: Acceptance letter

**DOI:** 10.1371/journal.pone.0356141.r006

**Authors:** Yufeng Zhou

PONE-D-26-16095R2

PLOS One

Dear Dr. Beermann,

I'm pleased to inform you that your manuscript has been deemed suitable for publication in PLOS One. Congratulations! Your manuscript is now being handed over to our production team.

At this stage, our production department will prepare your paper for publication. This includes ensuring the following:

* All references, tables, and figures are properly cited

* All relevant supporting information is included in the manuscript submission,

* There are no issues that prevent the paper from being properly typeset

You will receive further instructions from the production team, including instructions on how to review your proof when it is ready. Please keep in mind that we are working through a large volume of accepted articles, so please give us a few days to review your paper and let you know the next and final steps.

Lastly, if your institution or institutions have a press office, please let them know about your upcoming paper now to help maximize its impact. If they'll be preparing press materials, please inform our press team within the next 48 hours. Your manuscript will remain under strict press embargo until 2 pm Eastern Time on the date of publication. For more information, please contact onepress@plos.org.

You will receive an invoice from PLOS for your publication fee after your manuscript has reached the completed accept phase. If you receive an email requesting payment before acceptance or for any other service, this may be a phishing scheme. Learn how to identify phishing emails and protect your accounts at https://explore.plos.org/phishing.

If we can help with anything else, please email us at customercare@plos.org.

Thank you for submitting your work to PLOS One and supporting open access.

Kind regards,

PLOS ONE Editorial Office Staff

on behalf of

Dr. Yufeng Zhou

Academic Editor

PLOS One
